# Preoperative Serum Levels of Mesothelin in Patients with Colon Cancer

**DOI:** 10.1155/2014/161954

**Published:** 2014-11-13

**Authors:** Özgür Bostancı, Özgür Kemik, Ahu Kemik, Muharrem Battal, Uygar Demir, Sevim Purisa, Alpaslan Yavuz, Mehmet Mihmanlı

**Affiliations:** ^1^Şişli Hamidiye Etfal Training and Research Hospital General Surgery Clinic, Istanbul, Turkey; ^2^Department of General Surgery, Yuzuncu Yil University Medical Faculty, Van, Turkey; ^3^Department of Biochemistry, Istanbul University Cerrahpasa Medical Faculty, Istanbul, Turkey; ^4^Department of Biostatistics, Istanbul University Medical Faculty, Istanbul, Turkey; ^5^Department of Radiology, Yuzuncu Yil University Medical Faculty, Van, Turkey

## Abstract

*Background*. Screening for biochemical markers is important for diagnosing colon cancer. In this study, the reliability of serum mesothelin levels as a potential diagnostic and screening instrument was evaluated concerning colon cancer. *Methods*. Ninety-five patients who had undergone colonoscopic examination and who were diagnosed with colon cancer were included in the study. The serum mesothelin levels were measured with the ELISA kits and were evaluated in terms of significant difference when compared between colon cancer and control group. *Results*. Patients with colon cancer had significantly higher mesothelin serum levels (*P* < 0.001) than the control groups. We found significant associations between serum levels and tumor grade, perineural invasion, and vascular invasion (resp., *P* < 0.001). *Conclusion*. Evaluating the serum levels of mesothelin has a potential to detect and screen the colon cancer in affected patients. Our data suggest that mesothelin exhibits effects towards colon cancer and serves as a biomarker for this deadly disease.

## 1. Introduction

Colon cancer is one of the most common malignancies worldwide and represents a global health problem [1]. The pathogenesis of colon cancer generally consists of a staged progression from normal colonic mucosa to adenoma and finally to carcinoma over a period of 7–10 years [2, 3]. This consecutive progression over time allows for early recognition and treatment.

Late diagnosis is often blamed for a poor prognosis [4]. The classical noninvasive and invasive methods of screening modalities involve occult blood in stool testing, fecal immunochemical testing, double-contrast barium enema, flexible sigmoidoscopy, and colonoscopy [5]. Although some of these screening modalities have been reported to reduce the rate of malignancy or mortality, cancer-related mortality can be reduced by dissolving premalignant adenomas and early localized cancer prior to the development of more advanced stages. Therefore, it is necessary to effectively perform early screening, recognition, and follow-up monitoring of colon cancer.

Over the last few years, a focus of research has been on serum tumor markers. Studying “mesothelin” has been attempted in various malignancies [6–11], but there is one particularly important study on this topic in the literature [12].

Mesothelin is a 40 kDa cell surface glycoprotein. The mesothelin gene is located on chromosome 16p13.3 and encodes at least four protein products, including megakaryocyte potentiating factor (MPF) [13, 14] and three isoforms of mesothelin, which are variant 1 (mesothelin) [14], the currently uncharacterized variant 2 [15, 16], and variant 3 (soluble-mesothelin-related protein (SMRP)) [17].

MPF is a 31 kDa secreted cytokine-like protein that stimulates colony formation of mouse bone marrow cells in the presence of interleukin 3 [13, 14]. The three isoforms of mesothelin share an expansive N-terminal region. Mesothelin is a glycophosphatidylinositol- (GPI-) linked cell-surface glycoprotein of virtually 40 kDa and is thought to be a diversification molecule of the mesothelium [15]. Mesothelin can be present on or shed from the cell surface [18, 19]. SMRP in the GPI-anchor region is responsible for cell surface reinforcement, and it has a special C-terminal hydrophilic queue that may make the protein soluble [17].

SMRP suggests that mesothelin regulation could be linked to the intracellular signaling cascade triggered by ligand-independent activation of receptor tyrosine kinase.

There is a good correlation between laboratory findings of the sensibility and specificity of mesothelin for differentiating malignancies [20]. Our aim was to improve our understanding of the serious role of mesothelin levels in colon cancer patients.

## 2. Materials and Methods

### 2.1. Patients

We performed a multicentric and prospective study. This study was performed in accordance with institutional ethical guidelines and was approved by the Medical Ethics Committee of Sisli Hamidiye Etfal Research and Training Hospital in Istanbul, Turkey. All patients provided written informed consent. The number of patients was based on our kits. All samples were from adult patients (age 32–71 years). None of the patients had received any chemotherapy before tumor resection. Serum samples to determine the mesothelin levels were obtained just prior to surgery. The control subjects were noncancerous, age- and sex-matched volunteers who might have had colon cancer. No control subjects had any known history of tumors. The collected serum samples were stored at −80°C and then analyzed for mesothelin using an ELISA kit (from USCN Life Sciences Inc., Hubei, China).

### 2.2. Quantification of the Serum Mesothelin by ELISA

Sandwich ELISA test with a catalog number DMSLNO (Human Mesothelin Quantikine ELISA Kit) (R&D Systems) was used.

We prepared all indicators, standard dilutions, and samples as directed in the product manual. Each well was given 100 *μ*L of assay diluent. We added 200 *μ*L of conjugate to each well and incubated the samples at room temperature for 2 hours on the shaker before washing 4 times. Substrate solution (200 *μ*L) was added to each well; the samples were incubated at room temperature for 30 minutes on the bench top. Then, 50 *μ*L of stop solution was added to each well, and the samples were read at 450 nm within 30 minutes.

Sensitivity is 0.022 ng/mL. Assay range is 0.156–10 ng/mL.

### 2.3. Statistical Analyses

Shapiro Wilk normality test control and histogram charts were drawn. The data are given as the mean, standard deviation, median, min, and max. The frequencies and percentages were also included. The two groups with normally distributed variables according to an independent groups *t*-test were compared with the others using the Mann-Whitney *U* test. Age variables that differed between the 2 groups were evaluated by analysis of covariance significance of the mean. Three and four group comparisons of normally distributed variables and one-way ANOVA with Tukey HSD test for pairwise comparisons were performed. Others were evaluated with Kruskal-Wallis one-way analysis of variance. Then, the Bonferroni corrections for pairwise comparisons were evaluated with the Mann-Whitney *U* test (significance limit *P* < 0.0167 comparing three groups; four groups were comparable with *P* < 0.0083). The gender variables in the patients and controls were compared with the chi-square test. The minimum significance was taken as two-tailed *P* < 0.05. The analyses were performed using SPSS 21 program.

## 3. Results

**Figure 1:**
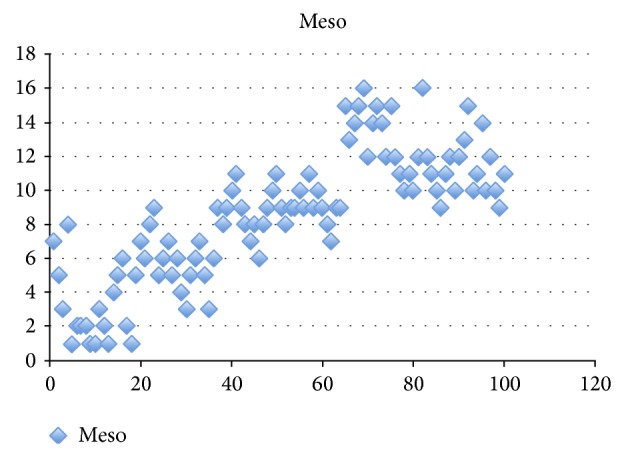
The distribution of mesothelin levels among patients was demonstrated.

The mean age of patients (59.9 years) was not significantly different from the mean age of the control group participants (58.7 years). Fifty-five percent of the controls were female and 58% of the patient group were female, and the difference was not significant (*P* > 0.05). The control subject and patient characteristics and their diagnoses are listed in Table 1. The mean serum mesothelin level in the control group was 0.19 ± 0.03 pg/mL. The mean serum mesothelin level in the patients with colon cancer was 8.47 ± 3.84 pg/mL and the distribution of mesothelin levels among patients was demonstrated by a graphical histogram in Figure 1. The serum mesothelin levels were significantly higher in the patients with colon cancer than in the control group (*P* < 0.0001) (Table 3). Age was homogeneous among the patients and controls (*P* = 0.281). Gender was similar between the patients and controls (*P* = 0.789).  Table 2 provides the serum mesothelin levels of patients with colon cancer according to clinicopathologic variables.

Table 2 provides the serum mesothelin levels of all patients and the control group. Twenty-four patients (24%) had tumor sizes of < 3 cm. Forty-one (43.1%) patients had distant metastasis. Twenty-five, 16, 21, and 38 patients had postoperative T stages of T1, T2, T3, and T4, respectively.

**Figure 2:**
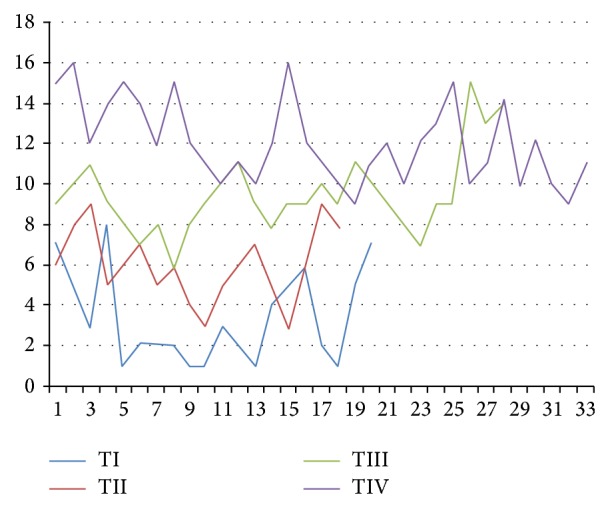
The disturbance of the serum mesothelin levels in terms of T stages among patients was demonstrated.

The serum mesothelin levels increased in T1 tumors to T4 tumors, and this difference was statistically significant (*P* < 0.001). The disturbance of the serum mesothelin levels in terms of T stages among patients was demonstrated in Figure 2. The minimum significance of bilateral comparisons with *P* < 0.0083 was included (due to the Bonferroni correction). Accordingly, all of the phases differed (*P* < 0.001). Additionally, the serum mesothelin levels were significantly higher in patients with colon cancer with increasing tumor size, lymph vascular involvement, distant metastasis, and lymph node metastasis (*P* < 0.001). A Bonferroni-corrected Mann-Whitney test was used for binary comparisons, and the limit of significance with *P* < 0.167 was included. Accordingly, all categories are different for each group (*P* < 0.001).

The tumor size, lymph vascular involvement, and distant metastasis were homogeneous with respect to the age variable between the groups (resp., *P* = 0.530, *P* = 0.701, *P* = 0.398, and *P* = 0.510). Lymph node metastases and distant metastases were similar to the gender variable between the groups (resp., *P* = 0.402, *P* = 0.719). Gender was distributed homogenously between the T stages (*P* = 0.831).

We observed a significant correlation between the serum mesothelin levels and tumor size with higher mesothelin levels detected for tumors > 3 cm in size (*P* = 0.003) as well as between the serum mesothelin levels and T stages (*P* = 0.002). The serum mesothelin levels were significantly correlated with distant metastasis, lymph node metastasis, and lymph vascular involvement (*P* = 0.004, *P* = 0.005, and *P* = 0.003).

## 4. Discussion

This analysis of 95 colon cancer patients indicated that serum mesothelin levels might be of predictive value in colon cancer, especially in the analysis of the clinical stage. For mesothelin, recent studies have primarily focused on the sensitivity and specificity of its diagnostic and early detection value for repetitions [6, 7, 11, 16, 17, 19].

The clinical stage is the most serious factor for the prognosis of colon cancer patients. Various systems can be used to classify colon cancer. Among them, the International Union Against Cancer's TNM staging is one of the most expansive. Although the TNM system has effectively classified patients based on their prognosis according to clinicopathological variables, it has reached a limit in providing critical information that may shape the treatment strategy.

Our study indicated that poor prognosis could be related to mesothelin levels based on the progression of the clinical advanced stage.

Although this study involved a small number of patients, the results for the association between the mesothelin levels and advanced colon cancer were statistically significant. Due to the small number of stage III and stage IV (*n* = 22 and *n* = 25) colon cancer patients, future investigations are needed to validate these findings.

Mechanisms that regulate MSLN transcription levels and mesothelin cell-surface expression or dismiss as a soluble form in patient fluids are not well understood. Several pathways have been explored. MSLN gene was found to be hypomethylated in pancreatic ductal adenocarcinoma, consistent with the inverse correlation between mRNA expression and DNA methylation described in numerous cancers. Also, mesothelin upregulation in carcinomas has been associated with a misregulation of Wnt signal transduction pathway. In mouse mammary epithelial cells, Wnt-5a downregulates mesothelin expression, possibly through antagonism of the Wnt/beta-catenin pathway, while in human colon cancer cells the enforcement expression of an N-terminal *β*-catenin binding site missing high mobility group- (HMG-) box T-cell factor 1 is associated with the upregulation of several GPI-anchored adhesion molecules, including mesothelin. Moreover, the overexpression of mesothelin in exon 9 GISTs suggests that mesothelin regulation could be linked to the intracellular signaling cascade detented by ligand-independent activation of receptor tyrosine kinase.

Mesothelin may behave as a clue reagent-marker of the intercellular pathway, leading to distant metastases and angiogenesis in colon cancer. The association of mesothelin with other signaling molecules and pathways must be approached with the goal of understanding the molecular pathogenesis of these tumors.

In conclusion, we observed a continuous correlation between the serum mesothelin levels and tumor metastasis in colon cancer. The valuation in the serum mesothelin levels, especially in patients with colon cancer, may have predictive potential and may also facilitate the development of treatment strategies for colon cancer patients.

## Figures and Tables

**Table 1:** The clinicopathologic features of patients and controls.

Patients		Controls	*P*
(*n* = 100)		(*n* = 70)
Age (y)	58.7 ± 3.9	59.9 ± 3.4	<0.05
Gender (M/F)	43/52	31/39	

Tumor size			
≤3 cm	20		
>3 cm	75		
TNM stage			
TI	21		
TII	17		
TIII	23		
TIV	34		
Invasion			
T1	20		
T2	15		
T3	23		
T4	37		
Lymph node metastasis			
N0	32		
N1	28		
N2	35		
Metastasis			
Present	70		
Absent	21		

**Table 2:** Preoperative serum mesothelin levels of the clinicopathologic variables of the patients (mean ± SD) (minimum–maximum) were indicated.

	Mesothelin (pg/mL)
Tumor size	
≤3 cm	5.02 ± 2.7 (1–9)
>3 cm	10.86 ± 2.42 (8–16)
TNM stage	
TI	3.4 ± 2.3 (1–8)
TII	6.0 ± 1.7 (3–9)
TIII	9.29 ± 1.8 (6–15)
TIV	12.09 ± 2.03 (9–16)
Invasion	
T1	3.11 ± 2.22 (1–8)
T2	5.72 ± 10.56 (3–9)
T3	8.89 ± 1.19 (6–11)
T4	12.19 ± 2.04 (9–16)
Lymph node metastasis	
N0	4.19 ± 2.57 (1–9)
N1	6.72 ± 2.34 (3–11)
N2	11.02 ± 2.38 (6–16)
Metastasis	
Present	9.81 ± 3.02 (3–16)
Not present	3.73 ± 2.4 (1–8)

**Table 3:** Preoperative serum mesothelin levels of all patients and controls (mean ± SD) (min–max) were indicated.

	Controls	Patients	*P*
Mesothelin	0.19 ± 0.03	8.47 ± 3.84	<0.0001
(pg/mL)	(0.159–0.274)	(1–16)	
